# Navigating Insurance Policies in the United States for Gender-affirming Surgery

**DOI:** 10.1097/GOX.0000000000002564

**Published:** 2019-12-11

**Authors:** Wess A. Cohen, Alexa M. Sangalang, Margaret M. Dalena, Haripriya S. Ayyala, Jonathan D. Keith

**Affiliations:** From the Division of Plastic and Reconstructive Surgery, Rutgers New Jersey Medical School, Newark, N.J.

## Abstract

Patients with gender dysphoria seeking to undergo gender affirmation surgery are often challenged by lack of insurance coverage. The authors aim to review gender affirmation surgery policies and to highlight discrepancies between qualifying criteria across top insurance companies in the United States.

**Methods:**

The top 3 insurance companies in each state within the United States were determined by market share. Each insurance policy was analyzed according to coverage for specific “top surgeries” and “bottom surgeries.” Policies were obtained from company-published data and phone calls placed to the insurance provider.

**Results:**

Of the total 150 insurance companies identified, policies related to gender- affirming surgery were found for 124. Coverage for gender-affirming surgery varies by insurance company, state, and procedure. Most insurance companies, 122 of 124 (98%), covered chest masculinization, but only 25 of 124 (20%) of insurance companies covered nipple-areola complex reconstruction. Additionally, 36 of 124 (29%) insurance companies covered chest feminization. Vaginoplasty is covered by 120 of 124 (97%) insurance companies. Despite high rates of vaginoplasty coverage, vulvoplasty is only covered by 26 of 124 (21%) insurance companies. Phalloplasty and metoidioplasty are covered by 118 of 124 (95%) and 115 of 124 (93%) of insurance companies, respectively. Slightly more than half, 75 of 124 (60%) insurance companies covered penile prosthesis.

**Conclusions:**

As gender-affirming surgery insurance coverage increases, the policies regarding them remain inconsistent. Standardized policies across insurance companies would further increase access to gender-affirming surgery.

## INTRODUCTION

Approximately 1.4 million adults and 150,000 teens in the United States identified as transgender as of 2015.^1^ Many of these individuals have been diagnosed with gender dysphoria—the distress that is caused by a discrepancy between a person’s gender identity and the gender they were assigned at birth. Transfemales (TFs) are individuals assigned male at birth who identify as female, whereas transmales are individuals assigned female at birth who identify as male. Transgender patients frequently endure lack of acceptance, harassment, and assault, likely contributing to depression rates as high as 62%, as compared to rates of 16% in the general population.^2^ Additionally, suicide rates have been cited to be as high as 45% in this population.^3^

Gender-affirming surgery can provide life-changing results for transgender patients and has been shown to significantly improve patients’ self-esteem and functioning.^4^ These surgeries range from chest wall contouring procedures, such as mastectomy and breast augmentation, to penile and neovaginal reconstruction, and have proven to be effective in treating gender dysphoria.^5,6^ Despite the profound positive impact gender affirmation surgery provides, insurance coverage has been historically limited.^7^ However, in 2014, Medicare and Medicaid lifted the 1981 exclusion of transition-related care, and in 2017 an addendum to the Affordable Care Act banned discrimination on the basis of gender identity.^8,9^ Since then, some private insurers have increased coverage for gender affirmation surgery.^10,11^

Historically, most patients undergoing gender-affirming surgery have been self-pay. However, sociopolitical changes and expanding health insurance coverage have led to an increased incidence of gender-affirming surgery.^6,12,13^ Despite this, more than half of patients within the past year were denied insurance coverage for gender-affirming surgeries.^14^ The World Professional Association for Transgender Health, a nonprofit, interdisciplinary professional and educational organization devoted to transgender health, has set guidelines for which surgeries should be deemed medically necessary. Nonetheless, insurance coverage remains fragmented, inconsistent, and unclear to navigate.

Uncertainty surrounding insurance coverage for gender-affirming surgery contributes to confusion for providers and patients. It is critical for both plastic surgeons and transgender patients to be aware of the various insurance policies and potential hurdles for gender affirmation surgeries. The ability to navigate insurance policies will dramatically improve access to care for a traditionally underserved community. The authors aim to provide an overview of the current coverage atmosphere in the United States for gender affirmation surgeries and to highlight the challenges when navigating insurance policies. Although surgeons who routinely perform these surgeries may be familiar with the results described, the vast majority of plastic surgeons will not be. Additionally, this is the first manuscript to compile national insurance data on commonly performed gender-affirming surgeries.

## METHODS

The top 3 private insurance companies of each state in the continental United States were determined by market share as published by the Kaiser Family Foundation, a nonprofit, nonpartisan organization. Insurance companies were stratified into large and small group insurance companies by Kaiser. Only large groups were used in this study and were defined as having 101 or more employees. Policies as of December 11, 2018, from each insurance company regarding gender-affirming surgery were then obtained either by company-published online data or via phone call inquiry. Procedures analyzed for top surgery coverage were mastectomy, breast augmentation, and nipple-areola complex (NAC) reconstruction. Bottom surgery analysis included penectomy, clitoroplasty, labiaplasty, vaginoplasty, vulvoplasty, vaginectomy, vulvectomy, phalloplasty, metoidioplasty, penile prosthesis, scrotoplasty, testicular prosthesis, and urethroplasty. Coverage was determined if the medical policy’s stated procedures were considered medically necessary and eligible for coverage. Exclusions were noted when the medical policy explicitly stated such procedures were not covered.

## RESULTS

**Fig. 1.**
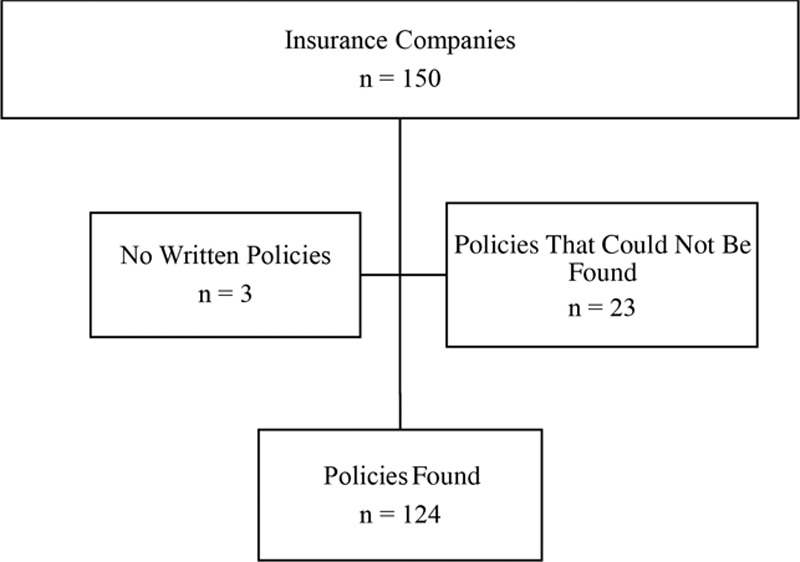
Insurance company inclusions and exclusions.

Coverage for gender-affirming surgery varies by insurance company, state, and procedure. Of the total 150 insurance companies identified, policies were found for 124. Three insurance companies had no written policy regarding gender affirmation surgery, and 23 insurance companies did not provide policy information after online and phone call inquiry (Fig. 1). Among the 123 insurance companies where policies were found, 3 of these companies stated that they cover genital surgery but did not specify which specific surgeries are included.

### Top Surgery

**Fig. 2.**
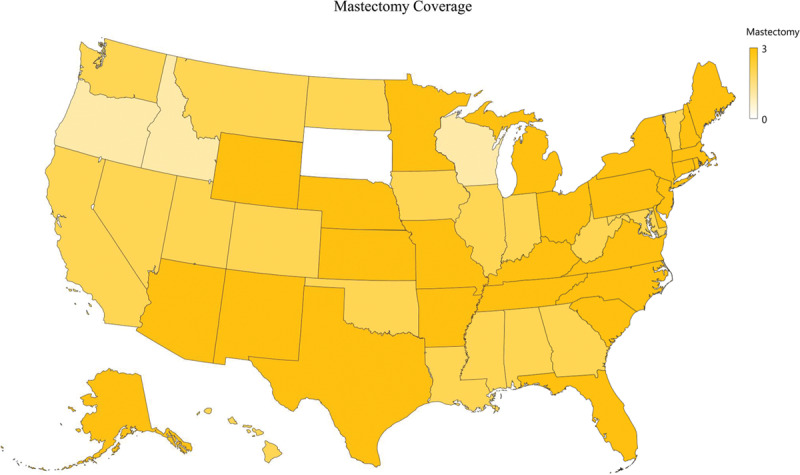
Chest masculinization coverage.

**Fig. 3.**
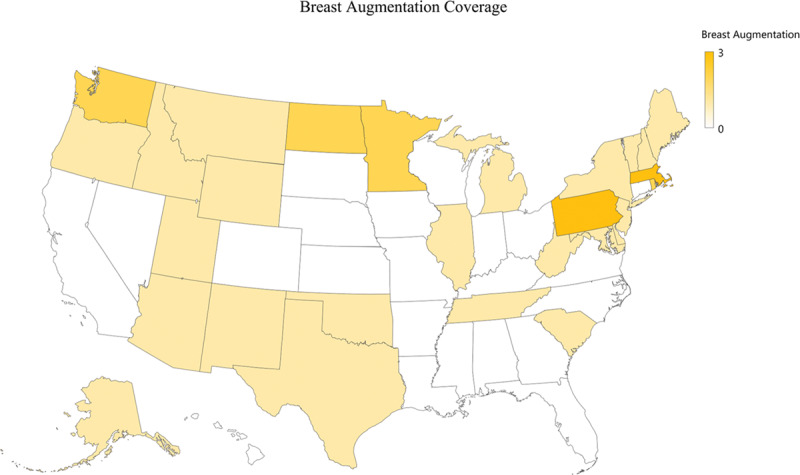
Chest feminization coverage.

**Fig. 4.**
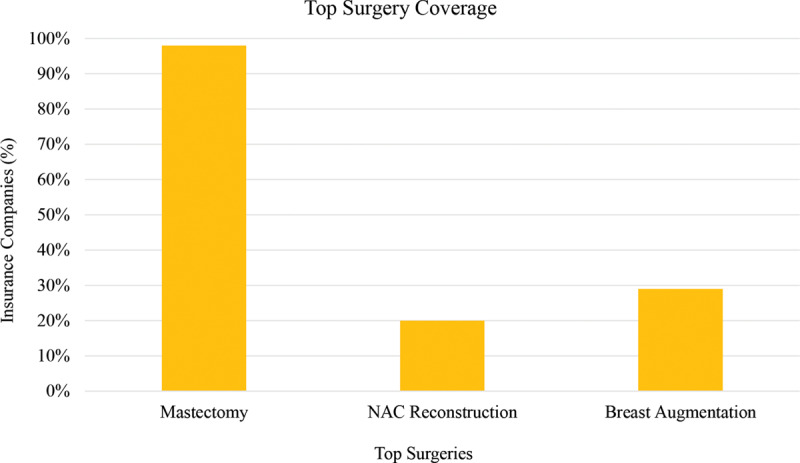
Insurance company coverage of top surgery.

Although most insurance companies, 122 of 124 (98%), covered mastectomy, 1 excluded mastectomy as medically necessary in the treatment of gender dysphoria (Fig. 2). Only 25 of 124 (20%) of insurance companies covered NAC reconstruction. 35 of 124 (28%) companies excluded NAC reconstruction coverage specifically. Only 36 of 124 (29%) insurance companies covered breast augmentation, whereas more than half, 84 of 124 (68%), of insurance companies deemed breast augmentation as not medically necessary (Figs. 3 and 4).

### Bottom Surgery: Male to Female

**Fig. 5.**
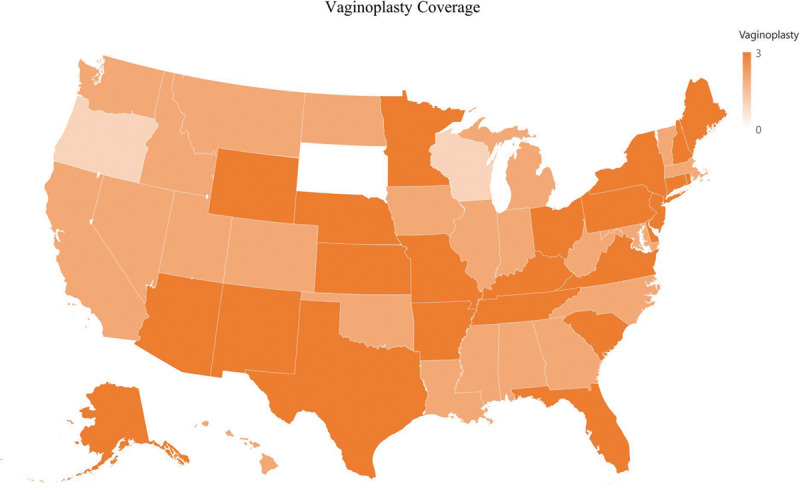
Vaginoplasty coverage.

**Fig. 6.**
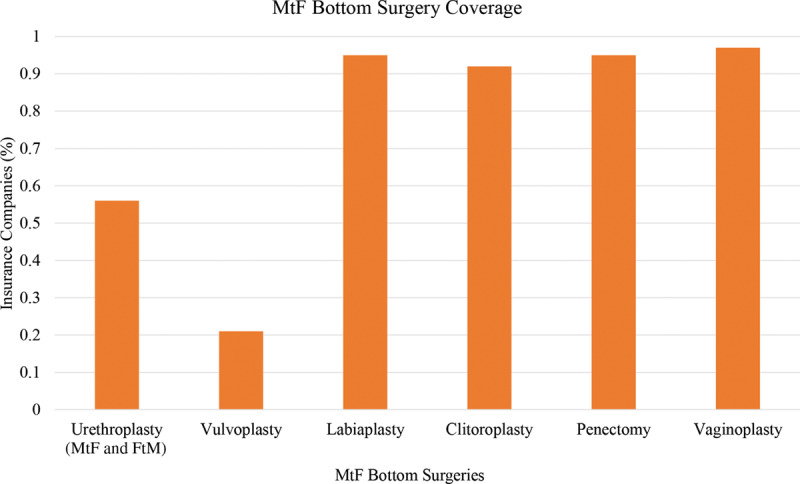
Bottom surgery: MtF.

Vaginoplasty is covered by 120 of 124 (97%) of insurance companies, and penectomy is covered by 118 of 124 (95%) insurance companies (Fig. 5). Additionally, clitoroplasty is covered by 114 of 124 (92%) companies and labiaplasty is covered by 116 of 124 (95%) of companies. Despite high rates of vaginoplasty coverage, vulvoplasty is only covered by 26 of 124 (21%) insurance companies (Fig. 6).

### Bottom Surgery: Female to Male

**Fig. 7.**
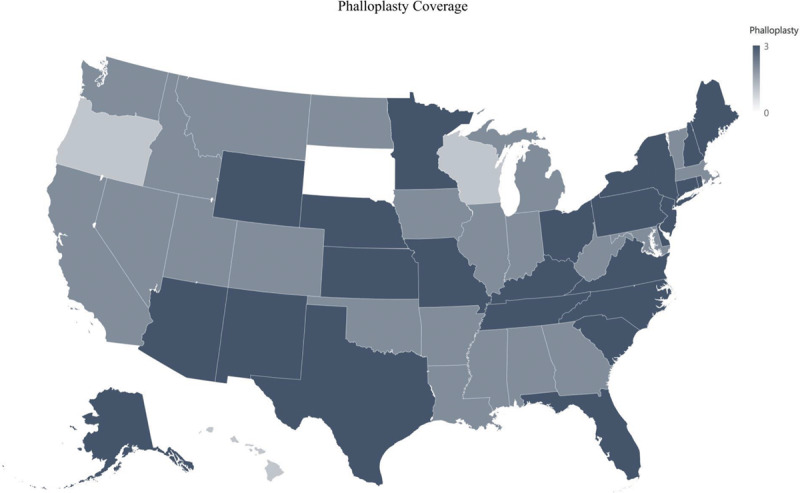
Phalloplasty coverage.

**Fig. 8.**
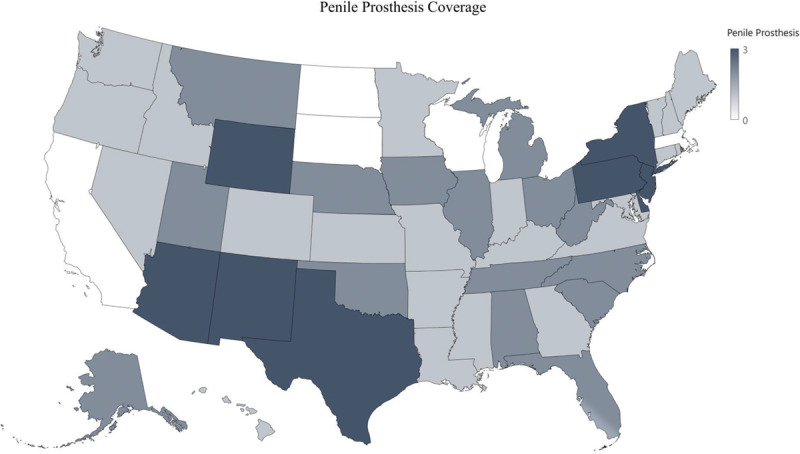
Penile prosthesis coverage.

**Fig. 9.**
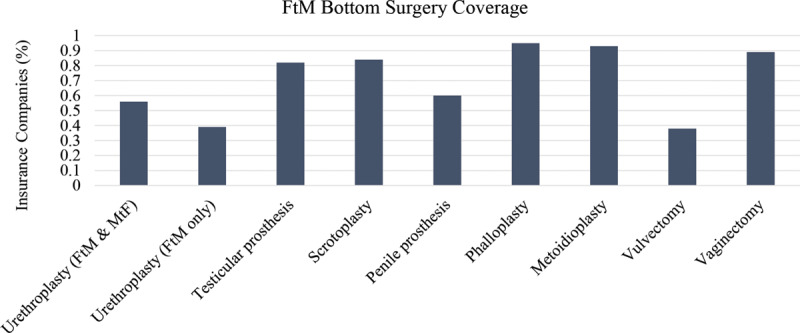
Bottom surgery: FtM.

Vaginectomy is covered by 110 of 124 (89%) of insurance companies; however vulvectomy is only covered by 47 of 124 (38%). Phalloplasty and metoidioplasty are covered by 118 of 124 (95%) and 115 of 124 (93%) of insurance companies, respectively (Fig. 7). Slightly more than half, 75 of 124 (60%) insurance companies covered penile prosthesis, and 7 (6%) insurance companies specifically excluded its coverage (Fig. 8). Scrotoplasty is covered by 104 of 124 (84%) of insurance companies; however, 7 (6%) insurance companies explicitly state its exclusion of coverage. One hundred two of 124 (82%) insurance companies covered testicular prosthesis, yet 10 of 124 (8%) of insurance companies excluded it. Although a total of 117 insurance companies covered urethroplasty, only 69 of these covered urethroplasty in both female-to-male (FtM) and male-to-female (MtF) gender affirmation surgery. The remaining 48 insurance companies only covered urethroplasty in FtM surgery (Fig. 9).

## DISCUSSION

Gender-affirming surgeries improve patient well-being, cosmesis, and sexual function.^15^ Unfortunately, financial burden is a frequently reported barrier to gender-affirming care.^16,17^ Transgender patients specifically encounter economic hardship with almost half earning less than $10,000 annually.^17^ Not coincidentally, gender-affirming surgery can improve a patient’s income which manifests a public good.^18^ Therefore, insurance coverage is critical for transgender patients seeking gender-affirming surgery. Despite these benefits, insurance coverage for gender-affirming surgery, while increasing, remains unreliable and vague.^19^ Although we did not observe geographic trends that correlated to a political map, the northeast and midatlantic regions trended toward broader coverage.

### Chest Masculinization

An overwhelming number of insurance companies covered FtM mastectomy. Breasts are a strong female-identifying characteristic,^20^ and therefore these patients often try to conceal their breasts either by wearing loose clothing or by binding their breasts, which may lead to skin damage, intertriginous infections, and even cellulitis.^21^ However, less than 20% of insurance companies covered NAC reconstruction, whereas another 25% implicitly exclude NAC reconstruction coverage (Fig. 4). This is most likely because MtF chest contouring is not done for oncologic reasons, and therefore the NAC does not necessarily need to be removed. However, the male nipple is located laterally and inferiorly as compared to the female nipple and not accounting for this by means of free nipple grafting may lead to unsatisfactory aesthetic results and may add to dysphoria.^22,23^

### Chest Feminization

Chest feminization was not deemed medically necessary by almost 75% of health insurers (Fig. 4). TF patients seek to solidify their feminine gender frequently through breast surgery. Although chest feminization significantly increases patient satisfaction, many insurance companies continue to consider breast augmentation equivalent to a cisgendered female desiring larger breasts and therefore consider it a cosmetic procedure. In fact, the current procedural terminology (CPT) code recognized by insurance companies is for bilateral augmentation mammoplasty with prosthetic implant: a traditional cosmetic code.^10^ However, when performing these procedures on TFs, it is reconstructive and should be covered by insurance. Coverage for breast implants may be further complicated by the inherent risks of placing a foreign body into a patient, which may lead to infection, capsular contracture, breast implant-associated anaplastic large cell lymphoma, cosmetic deformity, and need for additional procedures.^24^

### Bottom Surgery

The majority of insurance companies covered “bottom” surgeries. More than 90% of companies covered penectomies (Fig. 6). This is most likely because most health-care professionals believe that genitalia is what defines an individual’s sex.^25^ Moreover, if gender dysphoria is defined as discomfort or distress that is caused by a discrepancy between a person’s gender identity and that person’s sex assigned at birth,^26^ then bottom surgery can be considered a direct treatment.^17^

### Bottom Surgery: MtF

The associated procedures with penectomies for vaginal reconstruction, including clitoroplasty, labiaplasty, and vaginoplasty, were also covered by more than 90% of insurance companies (Fig. 6). This supports the idea that most professionals agree that the creation of the corresponding genitalia would inherently treat the dissociation between their gender identity and sex assigned at birth. Although a penectomy is the first step in constructing female genitalia, it is clear that most insurance companies believe that creating a functional vagina that can receive penetrative intercourse and shortening the urethra are important.

Interestingly, less than one-third of insurance companies covered a vulvoplasty, which is the creation of the external appearance of female genitalia without the creation of the vaginal canal (Fig. 6). This may be an option for patients who are older, have higher BMI, or have preexisting conditions such as prostatic radiation as the complication rate and risk profile is significantly lower than a vaginoplasty. Additionally, vulvoplasty was still associated with high levels of satisfaction.^27^ It is unclear if insurance companies consider this procedure “cosmetic” and therefore justify not covering it.

### Bottom Surgery: FtM

Similar to MtF bottom surgeries, the majority of FtM bottom surgeries were covered by insurance companies. Vaginectomy and related FtM bottom surgeries including phalloplasty and metoidioplasty were covered by more than 85% of companies (Fig. 9). Similar to penectomies, insurance companies agree that the removal of the genitals, ie, vaginectomy, can treat gender dysphoria. However, unlike MtF procedures, FtM procedures can also include procedures that increase function in addition to aesthetics such as penile prosthesis, which was covered by less than half of the insurance companies. Insurance companies may contend that phalloplasties without prosthesis already improve quality of life and sexual function^28^ and therefore penile prosthesis is not necessary. However, penile prosthesis with or without inflation could further increase sexual satisfaction by providing penetrative intercourse.^29,30^ Further studies are needed to delineate patient satisfaction with and without penile prosthesis.

Interestingly, more than 80% of companies cover a scrotoplasty and testicular prosthesis (Fig. 9). It is unclear why such a high proportion of insurance companies cover these nonfunctional procedures, but have chosen to forgo coverage of NAC reconstruction: similarly nonfunctional, but aesthetically native. The high rate of coverage of these procedures further demonstrates medical insurance companies possible opinion that genital surgery is a direct treatment for gender dysphoria, despite their lack of consistency regarding vulvoplasty. Urethroplasty is covered by more than 90% of insurance companies as it is necessary to lengthen the urethra when creating a neophallus to achieve normal micturition (Fig. 9).

### Criteria for Surgery

We encountered little consistency in which procedures insurance companies would cover, mirroring our own practice frustrations. The World Professional Association for Transgender Health provides standard-of-care guidelines and a list of surgical procedures that may be useful in treating MtF and FtM patients and is often utilized as a guide for insurance companies and health-care providers.^31^ However, we found little uniformity in criteria for coverage for any gender-affirming surgery within or between states. At a minimum, documentation of persistent gender dysphoria by a qualified mental health professional and further criteria including capacity, age of majority, no other significant medical or mental health problems, hormone therapy, and real-life experience may should be obtained.

### Future Directions

Although we were unable to deduce any geographic or insurance company trends to coverage, we believe this presents an opportunity for those performing gender-affirming surgeries to advocate for their patients. Surgeons will need to continue to communicate with one another, publish their results, and lobby the government as well as the insurance companies to expand coverage and increase transparency. Additionally, as the construct of “gender” continues to morph from binary to nonbinary and gender fluidity, gender-affirming surgeons must continue to understand their patients’ needs.

## CONCLUSIONS

As the demand for gender-affirming surgery continues to increase, it is critical for both the patient and physician to understand how to navigate insurance coverage policies. Greater awareness and transparency will improve access to care for a traditionally marginalized group of society. Additionally, more research is needed to delineate best practices for gender-affirming surgeries and their correlated patient-reported outcome measures.

## References

[1] Herman JL Flores AR Brown TNT Age of Individuals Who Identify as Transgender in the United States. 2017 Los Angeles, CA: The Williams Institute.

[2] Budge SL Adelson JL Howard KA Anxiety and depression in transgender individuals: the roles of transition status, loss, social support, and coping. J Consult Clin Psychol. 2013;81:545–557. 23398495 10.1037/a0031774

[3] Wolford-Clevenger C Cannon CJ Flores LY Suicide risk among transgender people: a prevalent problem in critical need of empirical and theoretical research. Violence Gend. 2017;4:69–72. 29062859 10.1089/vio.2017.0006 PMC5649411

[4] Hage JJ Karim RB Ought GIDNOS get nought? Treatment options for nontranssexual gender dysphoria. Plast Reconstr Surg. 2000;105:1222–1227. 10724285 10.1097/00006534-200003000-00063

[5] Padula WV Heru S Campbell JD Societal implications of health insurance coverage for medically necessary services in the U.S. Transgender population: a cost-effectiveness analysis. J Gen Intern Med. 2016;31:394–401. 26481647 10.1007/s11606-015-3529-6 PMC4803686

[6] Tran BNN Epstein S Singhal D Gender affirmation surgery: a synopsis using American College of Surgeons National Surgery Quality Improvement Program and National Inpatient Sample Databases. Ann Plast Surg. 2018;80 4 suppl 4):S229–S235. 29401127 10.1097/SAP.0000000000001350

[7] Canner JK Harfouch O Kodadek LM Temporal trends in gender-affirming surgery among transgender patients in the united states. JAMA Surg. 2018;153:609–616. 29490365 10.1001/jamasurg.2017.6231 PMC5875299

[8] Berli JU Knudson G Fraser L What surgeons need to know about gender confirmation surgery when providing care for transgender individuals: a review. JAMA Surg. 2017;152:394–400. 28196182 10.1001/jamasurg.2016.5549

[9] Samuels J Keisling M The anti-trans memo - abandoning doctors and patients. N Engl J Med. 2019;380:111–113. 30566376 10.1056/NEJMp1814406

[10] Weiss P Schechter L Coding for sex-reassignment surgery is evolving. Plast Surg News 2015;26:14.

[11] Ard KL Keuroghlian AS Training in sexual and gender minority health - expanding education to reach all clinicians. N Engl J Med. 2018;379:2388–2391. 30575482 10.1056/NEJMp1810522

[12] Liszewski W Peebles JK Yeung H Persons of nonbinary gender - awareness, visibility, and health disparities. N Engl J Med. 2018;379:2391–2393. 30575476 10.1056/NEJMp1812005 PMC6748626

[13] Baker KE The future of transgender coverage. N Engl J Med. 2017;376:1801–1804. 28402247 10.1056/NEJMp1702427

[14] James SE Herman JL Rankin S The Report of the 2015 U.S. Transgender Survey. Washington, DC 2016 National Center for Transgender Equality

[15] Papadopulos NA Lellé JD Zavlin D Quality of life and patient satisfaction following male-to-female sex reassignment surgery. J Sex Med. 2017;14:721–730. 28366591 10.1016/j.jsxm.2017.01.022

[16] Shipherd JC Green KE Abramovitz S Transgender clients: identifying and minimizing barriers to mental health treatment. J Gay Lesbian Ment Health. 2010;14:94–108.

[17] Puckett JA Cleary P Rossman K Barriers to gender-affirming care for transgender and gender nonconforming individuals. Sex Res Social Policy. 2018;15:48–59. 29527241 10.1007/s13178-017-0295-8 PMC5842950

[18] Emory LE Cole CM Avery E Client’s view of gender identity: Life, treatment status and outcome. 2003 18th Biennial Harry Benjamin Symposium, Gent, Belgium; Accessed 11 December 2018.

[19] Weissler JM Chang BL Carney MJ Gender-affirming surgery in persons with gender dysphoria. Plast Reconstr Surg. 2018;141:388e–396e. 10.1097/PRS.0000000000004123 29481407

[20] Kääriäinen M Salonen K Helminen M Chest-wall contouring surgery in female-to-male transgender patients: a one-center retrospective analysis of applied surgical techniques and results. Scand J Surg. 2017;106:74–79. 27107053 10.1177/1457496916645964

[21] Peitzmeier S Gardner I Weinand J Health impact of chest binding among transgender adults: a community-engaged, cross-sectional study. Cult Health Sex. 2017;19:64–75. 27300085 10.1080/13691058.2016.1191675

[22] Berry MG Curtis R Davies D Female-to-male transgender chest reconstruction: a large consecutive, single-surgeon experience. J Plast Reconstr Aesthet Surg. 2012;65:711–719. 22189204 10.1016/j.bjps.2011.11.053

[23] Lindsay WR Creation of a male chest in female transsexuals. Ann Plast Surg. 1979;3:39–46. 543632

[24] Weigert R Frison E Sessiecq Q Patient satisfaction with breasts and psychosocial, sexual, and physical well-being after breast augmentation in male-to-female transsexuals. Plast Reconstr Surg. 2013;132:1421–1429. 24281571 10.1097/01.prs.0000434415.70711.49

[25] The World Professional Association for Transgender Health. “Standards of care for the health of transsexual, transgender, and gender nonconforming people. 7th Version,” 2011 http://www.wpath.org.

[26] Fisk NM Editorial: gender dysphoria syndrome–the conceptualization that liberalizes indications for total gender reorientation and implies a broadly based multi-dimensional rehabilitative regimen. West J Med. 1974;120:386–391. 4839483 PMC1130142

[27] Jiang D Witten J Berli J Does depth matter? Factors affecting choice of vulvoplasty over vaginoplasty as gender-affirming genital surgery for transgender women. J Sex Med. 2018;15:902–906. 29706578 10.1016/j.jsxm.2018.03.085

[28] Murad MH Elamin MB Garcia MZ Hormonal therapy and sex reassignment: a systematic review and meta-analysis of quality of life and psychosocial outcomes. Clin Endocrinol (Oxf). 2010;72:214–231. 19473181 10.1111/j.1365-2265.2009.03625.x

[29] Morrison SD Chen ML Crane CN An overview of female-to-male gender-confirming surgery. Nat Rev Urol. 2017;14:486–500. 28508877 10.1038/nrurol.2017.64

[30] Morrison SD Massie JP Dellon AL Genital sensibility in the neophallus: getting a sense of the current literature and techniques. J Reconstr Microsurg. 2019;35:129–137. 30078177 10.1055/s-0038-1667360

[31] Schechter LS Safa B Introduction to phalloplasty. Clin Plast Surg. 2018;45:387–389. 29908627 10.1016/j.cps.2018.03.014

